# Supplementary material for: Predicting visual function by interpreting a neuronal wiring diagram
Source: Nature. 2024 Oct 2;634(8032):113–23. doi: 10.1038/s41586-024-07953-5 (PMC11446822; doi:10.1038/s41586-024-07953-5)
Supplement: Supplementary file 6 — CRF and ERF predictions for individual TmY4 and TmY9 cells. Analogous to Supplementary Data 3, but for TmY target types. Shown are the top four monosynaptic pathways, the strongest pathway passing through each of the top ten intermediary types (ranking from Extended Data Fig. 7), and the trisynaptic pathway Tm1–TmY–Dm3–TmY (see the section entitled Prediction of spatial normalization). [file 41586_2024_7953_MOESM6_ESM.zip › DataS4/TmY9q/720575940633731757.pdf]

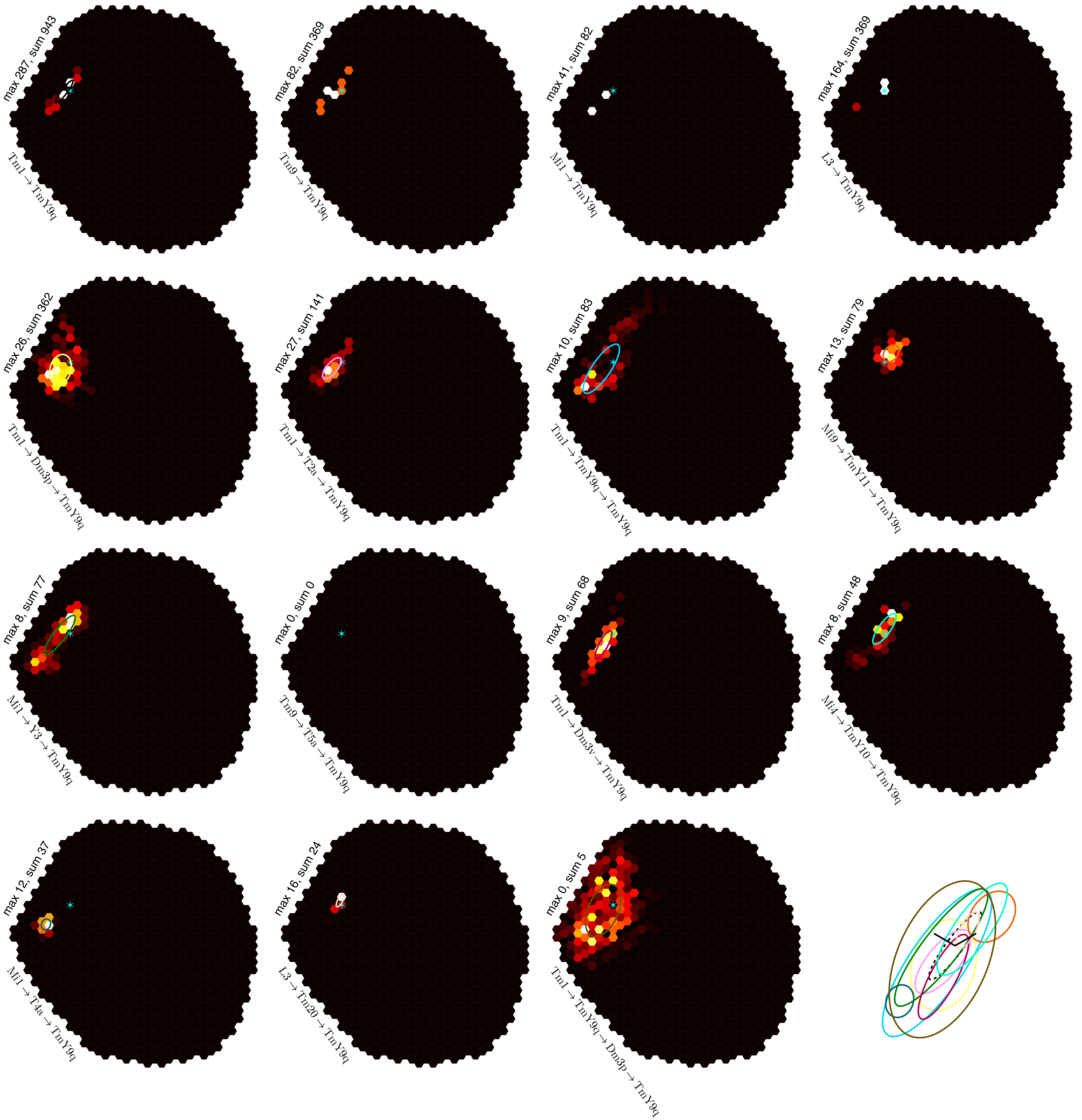
max 287, sum 943
Tm1 → TmY9q
max 82, sum 369
Tm9 → TmY9q
max 41, sum 82
Mi1 → TmY9q
max 164, sum 369
L3 → TmY9q
max 26, sum 362
Tm1 → Dm3p → TmY9q
max 27, sum 141
Tm1 → T2a → TmY9q
max 10, sum 83
Tm1 → TmY9q → TmY9q
max 13, sum 79
Mi9 → TmY11 → TmY9q
max 8, sum 77
Mi1 → Y3 → TmY9q
max 0, sum 0
Tm9 → T5a → TmY9q
max 9, sum 68
Tm1 → Dm3v → TmY9q
max 8, sum 48
Mi4 → TmY10 → TmY9q
max 12, sum 37
Mi1 → T4a → TmY9q
max 16, sum 24
L3 → Tm20 → TmY9q
max 0, sum 5
Tm1 → TmY9q → Dm3p → TmY9q
